# Development of an Eccentric Macular Hole Followed by Reopening of the Original Macular Hole as a Long-term Sequelae of Internal Limiting Membrane Peeling and Focal Laser Photocoagulation: A Case Report

**DOI:** 10.7759/cureus.44406

**Published:** 2023-08-30

**Authors:** Takashi Takeuchi, Hiromasa Hirai, Hironobu Jimura, Hiroki Tsujinaka, Nahoko Ogata, Tetsuo Ueda

**Affiliations:** 1 Ophthalmology, Nara Medical University, Kashihara, JPN

**Keywords:** laser photocoagulation, lens capsular transplantation, refractory macular hole, eccentric macular hole, internal limiting membrane peeling, pars plana vitrectomy

## Abstract

A macular hole (MH) is a widely known disease among ophthalmologists. Vitrectomy with internal limiting membrane (ILM) peeling is a standard technique for full-thickness MHs. However, the recurrence of MHs is sometimes seen. In addition, an eccentric MH is known to rarely occur after vitrectomy. An eccentric MH has been considered to require no therapeutic intervention because of its lack of increase in size. This study reports a case of two MHs (a recurrent MH and an enlarged eccentric MH) developed after laser photocoagulation around the injured retina caused by ILM peeling at the initial surgery.

A 56-year-old woman presented with an idiopathic MH in her left eye and best-corrected visual acuity (BCVA) was decreased to 20/80. She underwent phacoemulsification and vitrectomy combined with posterior hyaloid removal, ILM peeling, and 20% sulfur hexafluoride gas tamponade.

During the ILM peeling, we performed laser photocoagulation around the injured retina within the arcade. The MH was successfully closed and her BCVA was improved to 20/20 one month after surgery. Eight months after surgery, an eccentric MH occurred next to the photocoagulation spots. However, her BCVA remained 20/20; thus, we just followed up on her eye. Six years after surgery, her BCVA was decreased to 20/200. The eccentric MH increased in size and the original MH re-opened. The second vitrectomy was performed, but ILM had been already peeled within the arcade during the previous surgery and a usable sufficient size of ILM which could be auto-transplanted to the holes was not obtained. Thus, free flaps of the posterior lens capsule were harvested and placed within each hole. Two holes were successfully closed and her BCVA improved to 10/20 at three months after the surgery. Laser photocoagulation around the injured retina derived from ILM peeling may be a risk for recurrent MHs.

## Introduction

Pars plana vitrectomy (PPV) with posterior hyaloid removal, internal limiting membrane (ILM) peeling, and gas tamponade has been the standard treatment technique for full-thickness macular holes (MHs). However, the retina may be injured by microforceps when ILM peeling is attempted. Retinal damage is known to cause eccentric MHs, and several reports have reported secondary eccentric MHs after PPV [1-3]. Previous reports suggest that eccentric MH does not change in size over time and does not require therapeutic intervention. Additionally, the recurrence of MHs after PPV can be seen in several cases. The treatment of recurrent or persistent MH remains challenging for vitreoretinal surgeons because ILM had already been peeled in the previous surgery, and the ILM-free flap may not be readily available. Several reports have used lens capsular flap as a treatment for refractory MHs [4-7]. These studies have generally reported the treatment of a single MH, and treating multiple MHs has been rare. Herein, we report a case treated with autologous lens posterior capsule flaps for two MHs (a recurrent MH and an enlarged eccentric MH) developed after tight laser photocoagulation around the injured retina caused by ILM peeling at the initial surgery.

## Case presentation

**Figure 1:**
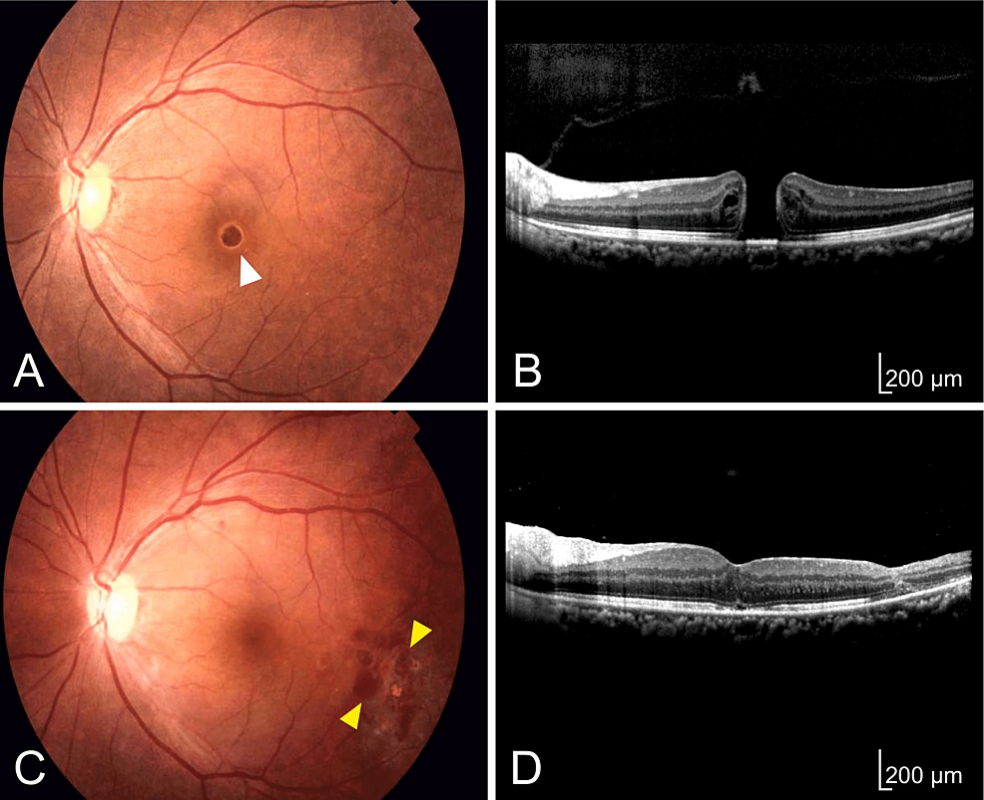
Initial examination of the left eye (A, B) and 10 days after the initial surgery (C, D). (A) Fundus photograph. The MH (white arrowhead) is open. (B) Optical coherence tomography (OCT). The OCT image displays the MH. The basal diameter of the MH is 771 μm. (C) Fundus photograph. The MH is closed. Several photocoagulation spots (yellow arrowheads) are detectable. (D) OCT. The OCT image presents a closed MH. The disturbance of the retinal layer structure at the temporal arcade is seen. MH, macular hole; OCT, optical coherence tomography; μm, micrometer.

A 56-year-old woman presented with central vision loss in her left eye for two weeks and visited our tertiary hospital (Nara Medical University Hospital). At the initial visit, her best-corrected visual acuity (BCVA) was 20/20 on the right and 20/80 on the left. The intraocular pressure was 15 millimeters of mercury in both eyes. The right was normal, but the left had an idiopathic MH (Figures 1A, 1B). She underwent PPV with posterior hyaloid removal, ILM peeling, and tamponade with 20% sulfur hexafluoride (SF6) gas combined with cataract surgery. The intraocular lens (IOL) used in this surgery was made of acrylic with a 7.0 mm optical diameter (Eternity X-70, SANTEN). During the ILM peeling, the microforceps pinched the retina near the macula several times, resulting in slight retinal trauma. Therefore, we performed laser photocoagulation around the traumatic area. The MH was closed, and her left BCVA improved to 20/20 one month after the surgery (Figures 1C, 1D). 

**Figure 2:**
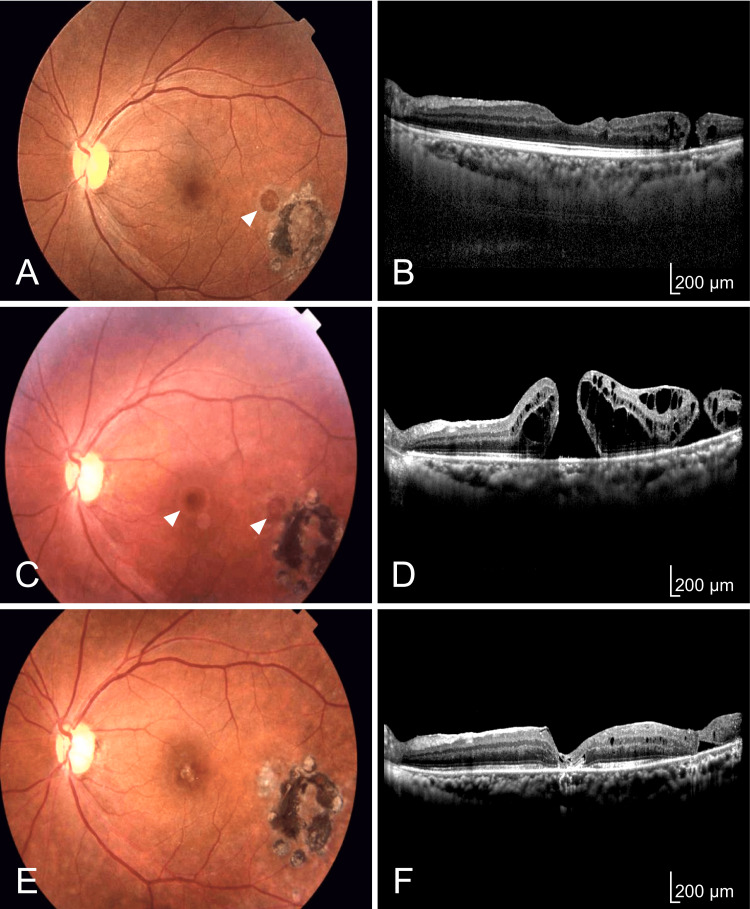
Eight months after the initial surgery (A, B), six years after the initial surgery (C, D), and three months after the second surgery (E, F). (A) Fundus photograph. An eccentric MH (white arrowhead) is discovered next to the photocoagulation scars. The photocoagulation scars are enlarged. (B) OCT. The OCT image presents an eccentric MH. The basal diameter of eccentric MH is 388 μm. (C) Fundus photograph. A recurrent MH and an eccentric MH (white arrowheads) are seen (D) OCT. The OCT image displays two holes. The basal diameter of recurrent MH is 1352 μm. The basal diameter of eccentric MH expands to 1019 μm. (E) Fundus photograph. The two holes are closed. (F) OCT. The OCT image confirms the closed holes. MH, macular hole; OCT, optical coherence tomography; μm, micrometer.

Eight months after the surgery, an eccentric MH was discovered next to the laser spots (Figures 2A, 2B), but her left BCVA remained 20/20. Thus, we continued regular follow-ups. Six years later, she presented with central vision loss and visited our hospital. Her left BCVA decreased to 20/200. The original MH re-opened, and the eccentric MH increased in size (Figures 2C, 2D). A second PPV was planned. However, we were concerned about the possibility that a sufficient measure of ILM, which could be auto-transplanted to the holes, might not be obtained. Therefore, we considered a plan of using a posterior lens capsule as an alternative. A single yttrium aluminum garnet (YAG) laser shot (1.0 mJ) was applied to the posterior lens capsule to create a slit on the day before the second surgery with the patient's consent. During the second surgery, we confirmed that the ILM had already been peeled within the arcade, and an ILM-free flap was not obtained. We decided to use the posterior lens capsule. We were able to obtain two flap pieces of posterior lens capsule by holding the created slit in the posterior capsule with microforceps and cutting them out. We had preoperatively identified the sizes of the two MHs (shown in the figure legend of Figure 2D). The flaps were trimmed to a size larger than the MHs and were placed within each hole with microforceps. After the flaps were manipulated to the proper position, viscoelastic material was placed onto the flaps to fix the position. Fluid-air exchange was performed with 20% SF6 gas. The patient was asked to maintain a head-down or prone position for one week. The two holes were closed, and her left BCVA improved to 10/20, three months after the surgery (Figures 2E, 2F).

## Discussion

Our case presented with two holes: a recurrent MH and an eccentric MH. The posterior lens capsular flaps were grafted to two MHs simultaneously, and successful closure was obtained in both. Recently, successful uses of lens capsules for MH have been reported [4-7]. Lens capsules for MH have been reported for both anterior and posterior capsules.

Similar to the ILM, the lens capsule is a basement membrane. It is thicker than the ILM but still possesses a pliable consistency. The lens capsule fills the entire MH and may provide a more substantial bridge for glial cell proliferation [6]. The advantage of a lens capsule flap is its higher density than the ILM, making it easier to settle down on the retinal surface and be directed to the designated place. In cases requiring cataract surgery, the anterior capsule may be easily secured through the continuous circular capsulorhexis, whereas the posterior capsule was easy to obtain with a pseudophakic eye. The IOL used in the first surgery was made of acrylic and had a 7.0 mm optical diameter. Therefore, the fundus visibility was good at the second surgery.

The ILM had already been peeled within the arcade, and we could not obtain a usable peripheral ILM in the case presented. Therefore, pieces of the posterior lens capsule were harvested due to her pseudophakic eye at the second surgery and transplanted into the two holes, successfully closed. There have been no reports of simultaneous closure of two macular holes, central and paracentral MHs, with the posterior capsule.

Our case also suggests that the combination of dense laser photocoagulation within the arcade and the slightly injured retina may have contributed to the enlargement of the eccentric MH and the recurrence of the original MH. The minor damage to the retina during the ILM peeling is widely known [8]. The injured retina has been proposed as a possible mechanism for eccentric MH [1]. In the present case, eccentric MH developed next to the injured retina where photocoagulations were performed. Previous reports have suggested that the size of the eccentric MH is stable and usually does not require further treatment [1,8,9]. However, in the present case, an eccentric MH increased in size over a long period (6 years postoperatively) and a recurrent MH appeared. Although few reports have discussed the relationship between eccentric MH and laser photocoagulation, Francone et al. have reported that photocoagulation around an eccentric MH was useful for lightening cystoid macular edema in patients with an eccentric MH developed after epiretinal membrane surgery [10]. This suggests that the laser around the eccentric MH may affect the macular structure and fluid circulation. In the present case, the macular structure might be fragile due to post-MH surgery, and the laser might have negatively affected the macular structure.

**Figure 3:**
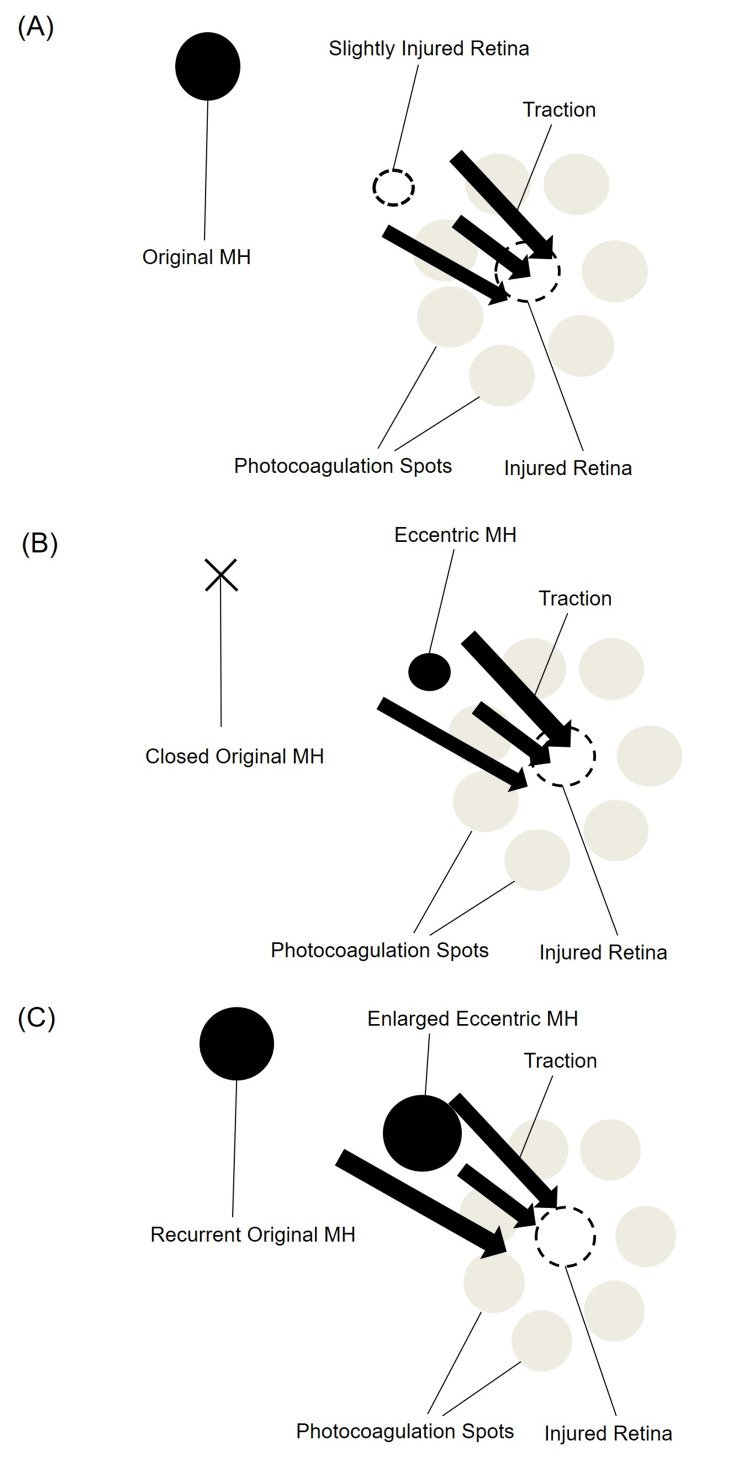
Diagrams of the mechanism considered in this case. (A) From the initial surgery to the development of the eccentric MH. (B) From the development of the eccentric MH to the recurrence of the original MH. (C) Completion of two MHs. MH, macular hole. Image credits: Hiromasa Hirai, Hironobu Jimura

We considered the following course in the present case. First, the microforceps held the retina within the arcade several times during the ILM peeling, making the slightly injured retina at the initial surgery. We performed dense laser photocoagulation around the injured area (Figure 3A). However, there was also a wounded area outside of the laser coagulation. Therefore, traction force was exerted on the area from the laser coagulation site, resulting in an eccentric MH (Figure 3B). The prolonged traction continued to act on the eccentric MH, leading to an increase in size and a recurrence of the original MH at the fovea (Figure 3C). Although we judged to perform laser photocoagulation around the injured retina at the initial surgery, the follow-up observation might have been preferable in this case.

## Conclusions

We reported a case of MHs leading to recurrent vision loss long after the initial surgery. During MH surgery, the highly dense laser photocoagulation around the injured retina within the arcade might result in eccentric MH and recurrent original MH. Although lens capsular transplantation was effective in this case, laser photocoagulation around the wounded retina during ILM peeling, which was close to the fovea, might require caution.
